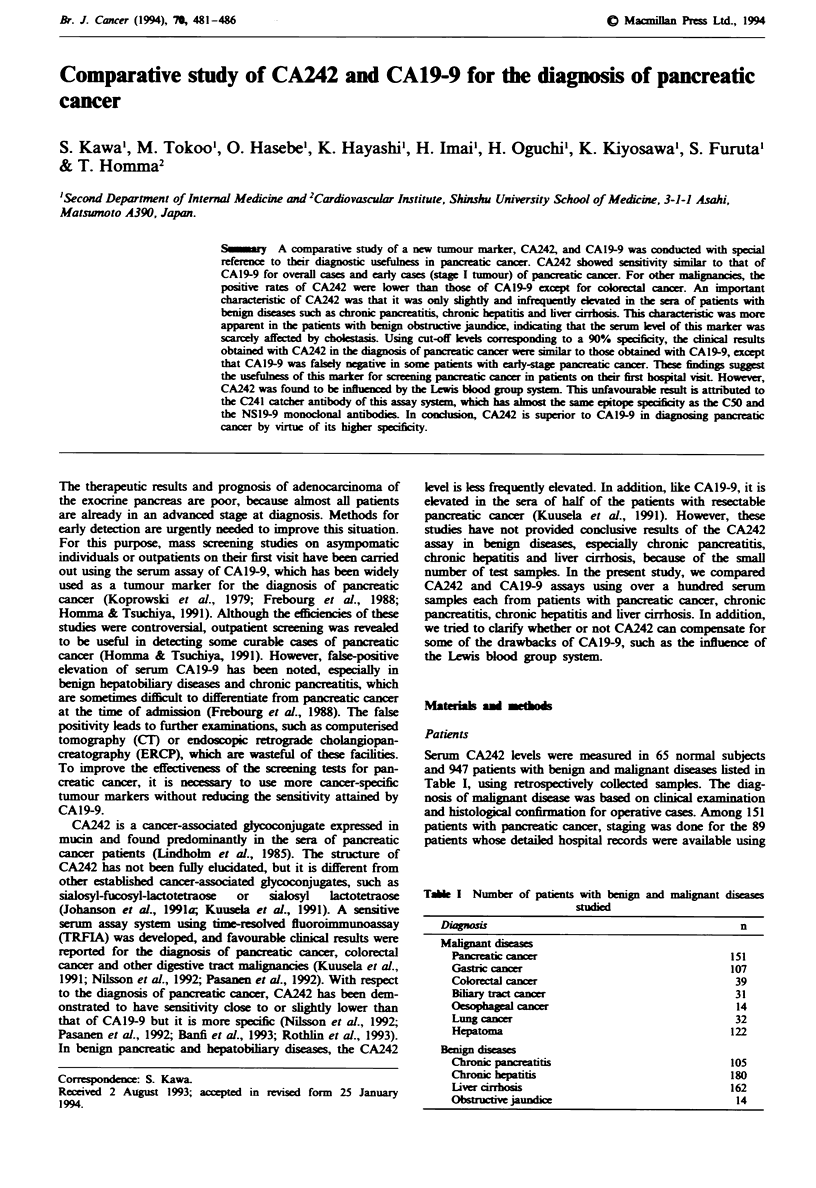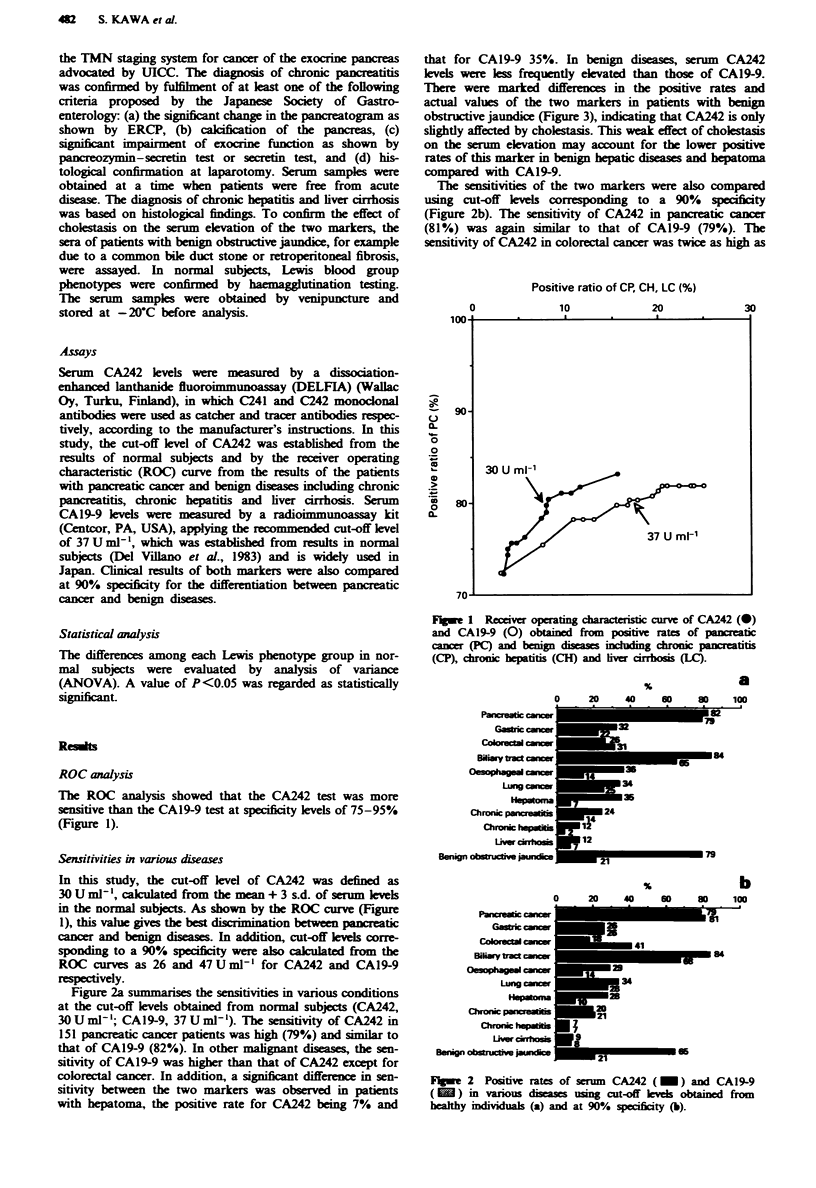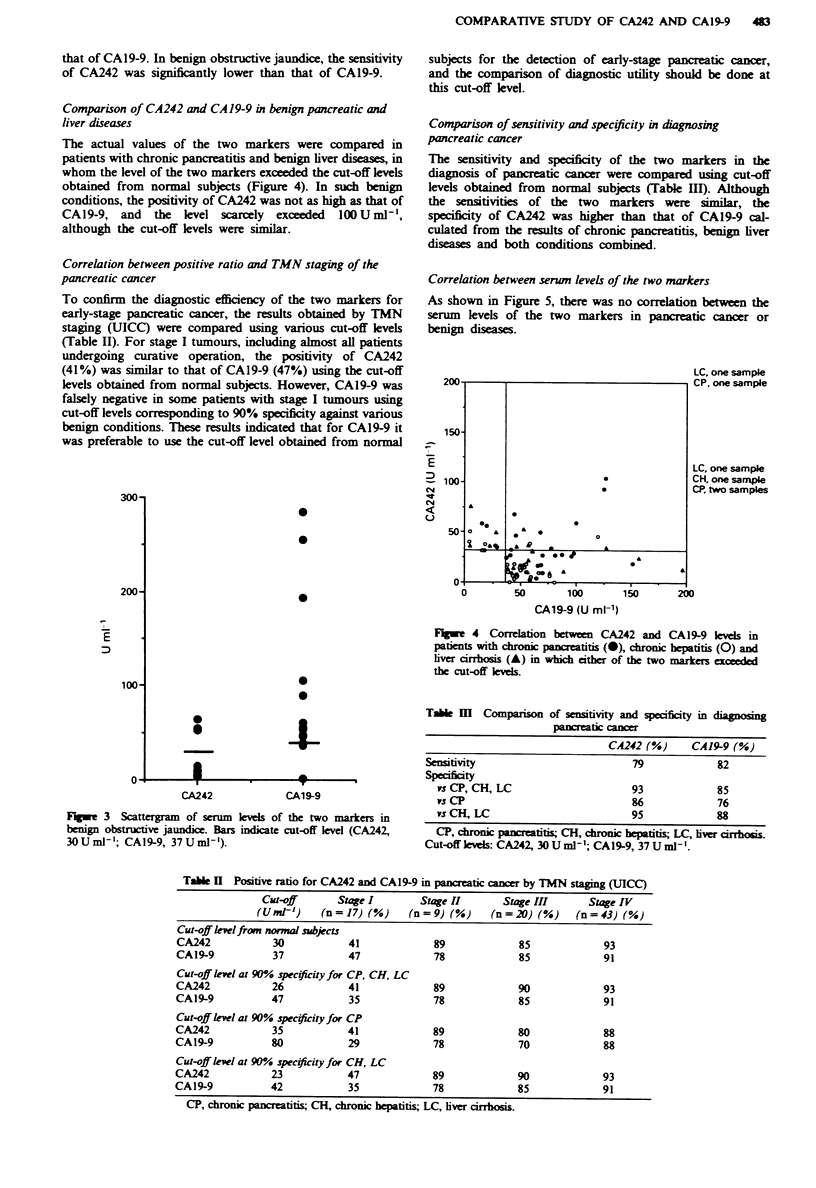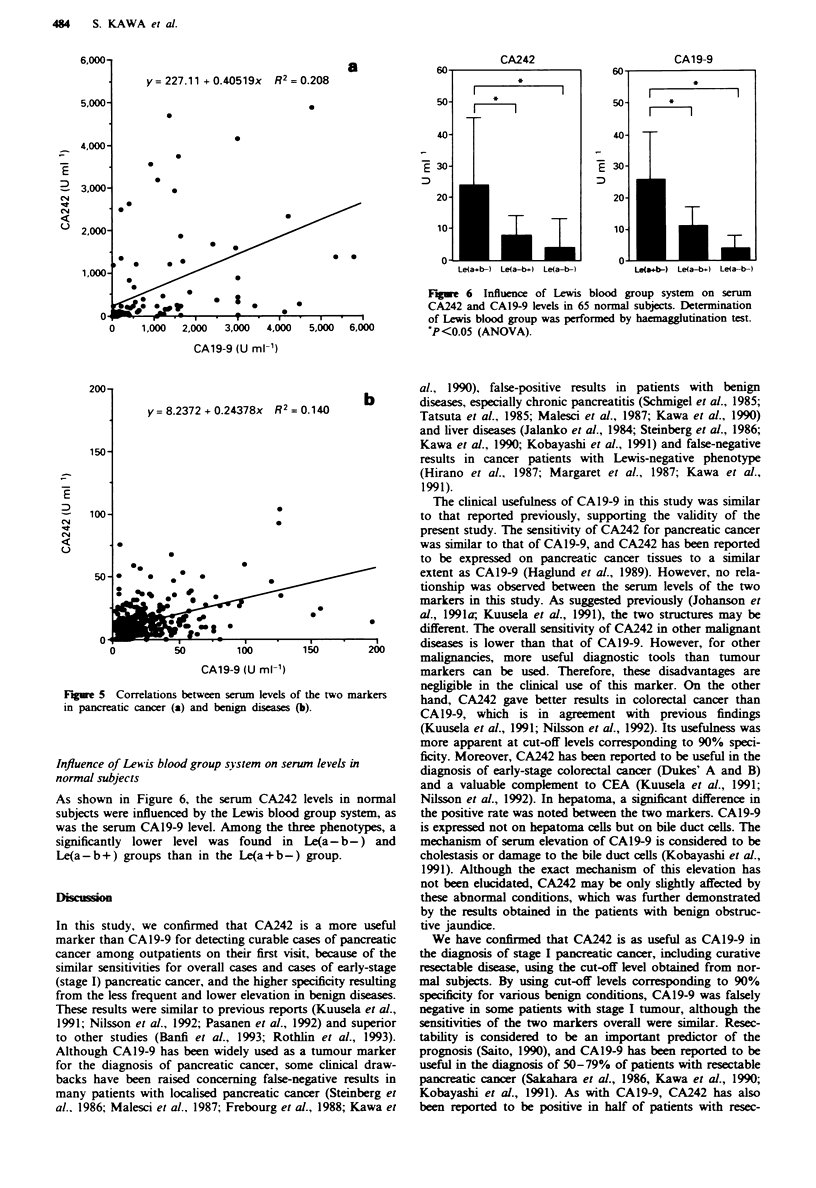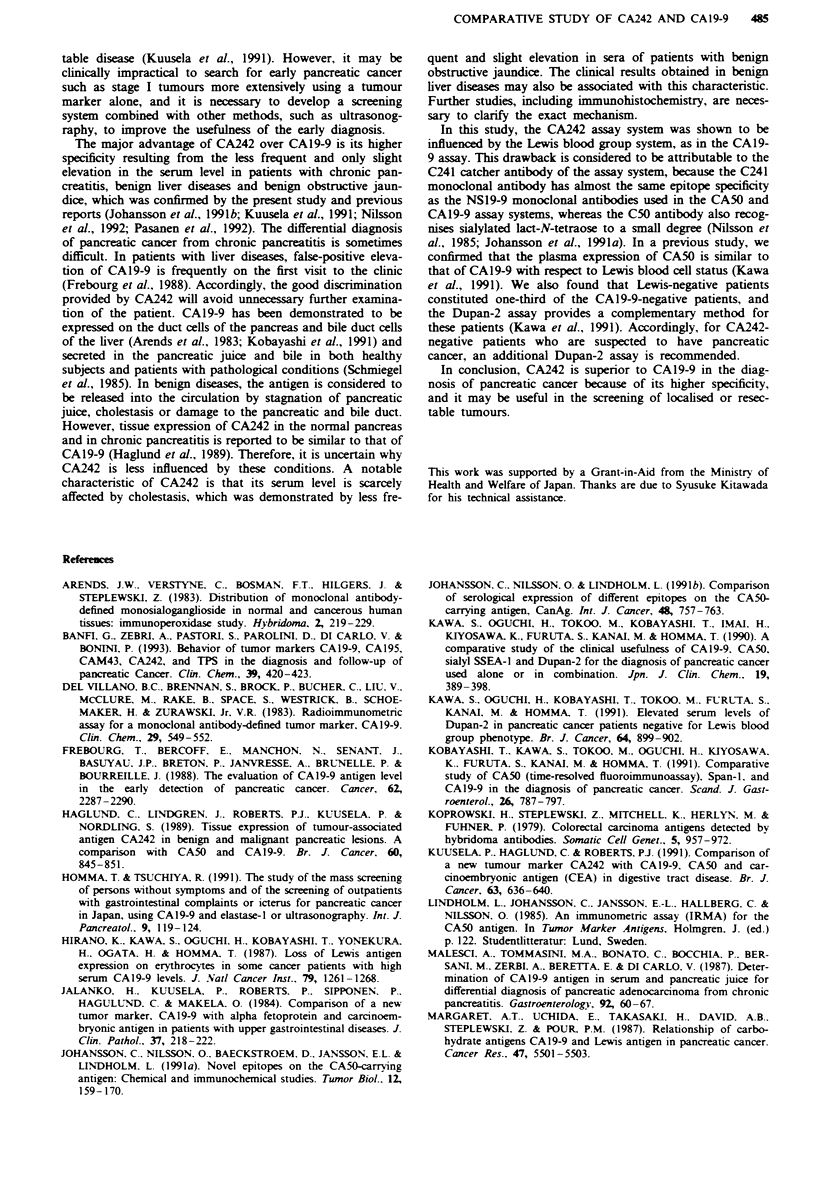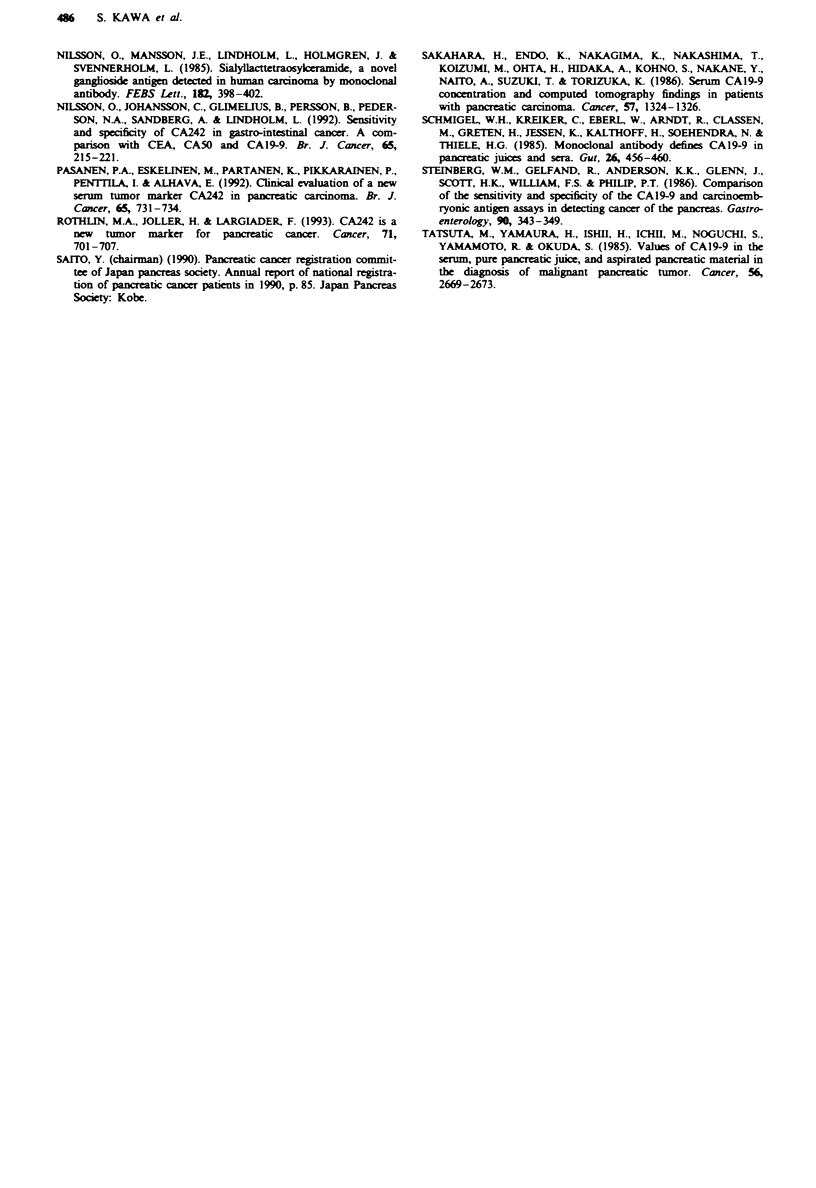# Comparative study of CA242 and CA19-9 for the diagnosis of pancreatic cancer.

**DOI:** 10.1038/bjc.1994.331

**Published:** 1994-09

**Authors:** S. Kawa, M. Tokoo, O. Hasebe, K. Hayashi, H. Imai, H. Oguchi, K. Kiyosawa, S. Furuta, T. Homma

**Affiliations:** Second Department of Internal Medicine, Shinshu University School of Medicine, Matsumoto, Japan.

## Abstract

A comparative study of a new tumour marker, CA242, and CA19-9 was conducted with special reference to their diagnostic usefulness in pancreatic cancer. CA242 showed sensitivity similar to that of CA19-9 for overall cases and early cases (stage I tumour) of pancreatic cancer. For other malignancies, the positive rates of CA242 were lower than those of CA19-9 except for colorectal cancer. An important characteristics of CA242 was that it was only slightly and infrequently elevated in the sera of patients with benign diseases such as chronic pancreatitis, chronic hepatitis and liver cirrhosis. This characteristic was more apparent in the patients with benign obstructive jaundice, indicating that the serum level of this marker was scarcely affected by cholestasis. Using cut-off levels corresponding to a 90% specificity, the clinical results obtained with CA242 in the diagnosis of pancreatic cancer were similar to those obtained with CA19-9, except that CA19-9 was falsely negative in some patients with early-stage pancreatic cancer. These findings suggest the usefulness of this marker for screening pancreatic cancer in patients on their first hospital visit. However, CA242 was found to be influenced by the Lewis blood group system. This unfavourable result is attributed to the C241 catcher antibody of this assay system, which has almost the same epitope specificity as the C50 and the NS19-9 monoclonal antibodies. In conclusion, CA242 is superior to CA19-9 in diagnosing pancreatic cancer by virtue of its higher specificity.


Br. J. Cancer (1994), 70, 481-486 © Macmillan Press Ltd., 1994

### Comparative study of CA242 and CA19-9 for the diagnosis of pancreatic cancer

S. Kawa[1], M. Tokoo[1], O. Hasebe[1], K. Hayashi[1], H. Imai[1], H. Oguchi[1], K. Kiyosawa[1], S. Furuta[1] & T. Homma[2]

[1]*Second Department of Internal Medicine and* [2]*Cardiovascular Institute, Shinshu University School of Medicine, 3-1-1 Asahi, Matsumoto A390, Japan.*

**Summary** A comparative study of a new tumour marker, CA242, and CA19-9 was conducted with special reference to their diagnostic usefulness in pancreatic cancer. CA242 showed sensitivity similar to that of CA19-9 for overall cases and early cases (stage I tumour) of pancreatic cancer. For other malignancies, the positive rates of CA242 were lower than those of CA19-9 except for colorectal cancer. An important characteristic of CA242 was that it was only slightly and infrequently elevated in the sera of patients with benign diseases such as chronic pancreatitis, chronic hepatitis and liver cirrhosis. This characteristic was more apparent in the patients with benign obstructive jaundice, indicating that the serum level of this marker was scarcely affected by cholestasis. Using cut-off levels corresponding to a 90% specificity, the clinical results obtained with CA242 in the diagnosis of pancreatic cancer were similar to those obtained with CA19-9, except that CA19-9 was falsely negative in some patients with early-stage pancreatic cancer. These findings suggest the usefulness of this marker for screening pancreatic cancer in patients on their first hospital visit. However, CA242 was found to be influenced by the Lewis blood group system. This unfavourable result is attributed to the C241 catcher antibody of this assay system, which has almost the same epitope specificity as the C50 and the NS19-9 monoclonal antibodies. In conclusion, CA242 is superior to CA19-9 in diagnosing pancreatic cancer by virtue of its higher specificity.

The therapeutic results and prognosis of adenocarcinoma of the exocrine pancreas are poor, because almost all patients are already in an advanced stage at diagnosis. Methods for early detection are urgently needed to improve this situation. For this purpose, mass screening studies on asympomatic individuals or outpatients on their first visit have been carried out using the serum assay of CA19-9, which has been widely used as a tumour marker for the diagnosis of pancreatic cancer (Koprowski *et al.*, 1979; Frebourg *et al.*, 1988; Homma & Tsuchiya, 1991). Although the efficiencies of these studies were controversial, outpatient screening was revealed to be useful in detecting some curable cases of pancreatic cancer (Homma & Tsuchiya, 1991). However, false-positive elevation of serum CA19-9 has been noted, especially in benign hepatobiliary diseases and chronic pancreatitis, which are sometimes difficult to differentiate from pancreatic cancer at the time of admission (Frebourg *et al.*, 1988). The false positivity leads to further examinations, such as computerised tomography (CT) or endoscopic retrograde cholangiopancreatography (ERCP), which are wasteful of these facilities. To improve the effectiveness of the screening tests for pancreatic cancer, it is necessary to use more cancer-specific tumour markers without reducing the sensitivity attained by CA19-9.

CA242 is a cancer-associated glycoconjugate expressed in mucin and found predominantly in the sera of pancreatic cancer patients (Lindholm *et al.*, 1985). The structure of CA242 has not been fully elucidated, but it is different from other established cancer-associated glycoconjugates, such as sialosyl-fucosyl-lactotetraose or sialosyl lactotetraose (Johanson *et al.*, 1991*a*; Kuusela *et al.*, 1991). A sensitive serum assay system using time-resolved fluoroimmunoassay (TRFIA) was developed, and favourable clinical results were reported for the diagnosis of pancreatic cancer, colorectal cancer and other digestive tract malignancies (Kuusela *et al.*, 1991; Nilsson *et al.*, 1992; Pasanen *et al.*, 1992). With respect to the diagnosis of pancreatic cancer, CA242 has been demonstrated to have sensitivity close to or slightly lower than that of CA19-9 but it is more specific (Nilsson *et al.*, 1992; Pasanen *et al.*, 1992; Banfi *et al.*, 1993; Rothlin *et al.*, 1993). In benign pancreatic and hepatobiliary diseases, the CA242 level is less frequently elevated. In addition, like CA19-9, it is elevated in the sera of half of the patients with resectable pancreatic cancer (Kuusela *et al.*, 1991). However, these studies have not provided conclusive results of the CA242 assay in benign diseases, especially chronic pancreatitis, chronic hepatitis and liver cirrhosis, because of the small number of test samples. In the present study, we compared CA242 and CA19-9 assays using over a hundred serum samples each from patients with pancreatic cancer, chronic pancreatitis, chronic hepatitis and liver cirrhosis. In addition, we tried to clarify whether or not CA242 can compensate for some of the drawbacks of CA19-9, such as the influence of the Lewis blood group system.

Correspondence: S. Kawa.
Received 2 August 1993; accepted in revised form 25 January 1994.

### Materials and methods

### *Patients*

Serum CA242 levels were measured in 65 normal subjects and 947 patients with benign and malignant diseases listed in Table I, using retrospectively collected samples. The diagnosis of malignant disease was based on clinical examination and histological confirmation for operative cases. Among 151 patients with pancreatic cancer, staging was done for the 89 patients whose detailed hospital records were available using

**Table I** Number of patients with benign and malignant diseases studied

| *Diagnosis* | n |
|---|---|
| Malignant diseases | |
| Pancreatic cancer | 151 |
| Gastric cancer | 107 |
| Colorectal cancer | 39 |
| Biliary tract cancer | 31 |
| Oesophageal cancer | 14 |
| Lung cancer | 32 |
| Hepatoma | 122 |
| Benign diseases | |
| Chronic pancreatitis | 105 |
| Chronic hepatitis | 180 |
| Liver cirrhosis | 162 |
| Obstructive jaundice | 14 |

the TMN staging system for cancer of the exocrine pancreas advocated by UICC. The diagnosis of chronic pancreatitis was confirmed by fulfilment of at least one of the following criteria proposed by the Japanese Society of Gastroenterology: (a) the significant change in the pancreatogram as shown by ERCP, (b) calcification of the pancreas, (c) significant impairment of exocrine function as shown by pancreozymin-secretin test or secretin test, and (d) histological confirmation at laparotomy. Serum samples were obtained at a time when patients were free from acute disease. The diagnosis of chronic hepatitis and liver cirrhosis was based on histological findings. To confirm the effect of cholestasis on the serum elevation of the two markers, the sera of patients with benign obstructive jaundice, for example due to a common bile duct stone or retroperitoneal fibrosis, were assayed. In normal subjects, Lewis blood group phenotypes were confirmed by haemagglutination testing. The serum samples were obtained by venipuncture and stored at −20°C before analysis.

### *Assays*

Serum CA242 levels were measured by a dissociation-enhanced lanthanide fluoroimmunoassay (DELFIA) (Wallac Oy, Turku, Finland), in which C241 and C242 monoclonal antibodies were used as catcher and tracer antibodies respectively, according to the manufacturer's instructions. In this study, the cut-off level of CA242 was established from the results of normal subjects and by the receiver operating characteristic (ROC) curve from the results of the patients with pancreatic cancer and benign diseases including chronic pancreatitis, chronic hepatitis and liver cirrhosis. Serum CA19-9 levels were measured by a radioimmunoassay kit (Centcor, PA, USA), applying the recommended cut-off level of 37 U $ml^{-1}$, which was established from results in normal subjects (Del Villano *et al.*, 1983) and is widely used in Japan. Clinical results of both markers were also compared at 90% specificity for the differentiation between pancreatic cancer and benign diseases.

### *Statistical analysis*

The differences among each Lewis phenotype group in normal subjects were evaluated by analysis of variance (ANOVA). A value of $P<0.05$ was regarded as statistically significant.

### Results

### *ROC analysis*

The ROC analysis showed that the CA242 test was more sensitive than the CA19-9 test at specificity levels of 75–95% (Figure 1).

### *Sensitivities in various diseases*

In this study, the cut-off level of CA242 was defined as 30 U $ml^{-1}$, calculated from the mean + 3 s.d. of serum levels in the normal subjects. As shown by the ROC curve (Figure 1), this value gives the best discrimination between pancreatic cancer and benign diseases. In addition, cut-off levels corresponding to a 90% specificity were also calculated from the ROC curves as 26 and 47 U $ml^{-1}$ for CA242 and CA19-9 respectively.

Figure 2a summarises the sensitivities in various conditions at the cut-off levels obtained from normal subjects (CA242, 30 U $ml^{-1}$; CA19-9, 37 U $ml^{-1}$). The sensitivity of CA242 in 151 pancreatic cancer patients was high (79%) and similar to that of CA19-9 (82%). In other malignant diseases, the sensitivity of CA19-9 was higher than that of CA242 except for colorectal cancer. In addition, a significant difference in sensitivity between the two markers was observed in patients with hepatoma, the positive rate for CA242 being 7% and that for CA19-9 35%. In benign diseases, serum CA242 levels were less frequently elevated than those of CA19-9. There were marked differences in the positive rates and actual values of the two markers in patients with benign obstructive jaundice (Figure 3), indicating that CA242 is only slightly affected by cholestasis. This weak effect of cholestasis on the serum elevation may account for the lower positive rates of this marker in benign hepatic diseases and hepatoma compared with CA19-9.

The sensitivities of the two markers were also compared using cut-off levels corresponding to a 90% specificity (Figure 2b). The sensitivity of CA242 in pancreatic cancer (81%) was again similar to that of CA19-9 (79%). The sensitivity of CA242 in colorectal cancer was twice as high as

**Figure 1** Receiver operating characteristic curve of CA242 (●) and CA19-9 (○) obtained from positive rates of pancreatic cancer (PC) and benign diseases including chronic pancreatitis (CP), chronic hepatitis (CH) and liver cirrhosis (LC).

**Figure 2** Positive rates of serum CA242 (■) and CA19-9 (▨) in various diseases using cut-off levels obtained from healthy individuals (**a**) and at 90% specificity (**b**).

that of CA19-9. In benign obstructive jaundice, the sensitivity of CA242 was significantly lower than that of CA19-9.

*Comparison of CA242 and CA19-9 in benign pancreatic and liver diseases*

The actual values of the two markers were compared in patients with chronic pancreatitis and benign liver diseases, in whom the level of the two markers exceeded the cut-off levels obtained from normal subjects (Figure 4). In such benign conditions, the positivity of CA242 was not as high as that of CA19-9, and the level scarcely exceeded $100\,U\,ml^{-1}$, although the cut-off levels were similar.

*Correlation between positive ratio and TMN staging of the pancreatic cancer*

To confirm the diagnostic efficiency of the two markers for early-stage pancreatic cancer, the results obtained by TMN staging (UICC) were compared using various cut-off levels (Table II). For stage I tumours, including almost all patients undergoing curative operation, the positivity of CA242 (41%) was similar to that of CA19-9 (47%) using the cut-off levels obtained from normal subjects. However, CA19-9 was falsely negative in some patients with stage I tumours using cut-off levels corresponding to 90% specificity against various benign conditions. These results indicated that for CA19-9 it was preferable to use the cut-off level obtained from normal subjects for the detection of early-stage pancreatic cancer, and the comparison of diagnostic utility should be done at this cut-off level.

*Comparison of sensitivity and specificity in diagnosing pancreatic cancer*

The sensitivity and specificity of the two markers in the diagnosis of pancreatic cancer were compared using cut-off levels obtained from normal subjects (Table III). Although the sensitivities of the two markers were similar, the specificity of CA242 was higher than that of CA19-9 calculated from the results of chronic pancreatitis, benign liver diseases and both conditions combined.

*Correlation between serum levels of the two markers*

As shown in Figure 5, there was no correlation between the serum levels of the two markers in pancreatic cancer or benign diseases.

**Figure 3** Scattergram of serum levels of the two markers in benign obstructive jaundice. Bars indicate cut-off level (CA242, $30\,U\,ml^{-1}$; CA19-9, $37\,U\,ml^{-1}$).

**Figure 4** Correlation between CA242 and CA19-9 levels in patients with chronic pancreatitis (●), chronic hepatitis (O) and liver cirrhosis (▲) in which either of the two markers exceeded the cut-off levels.

**Table III** Comparison of sensitivity and specificity in diagnosing pancreatic cancer

| | *CA242 (%)* | *CA19-9 (%)* |
|---|---|---|
| Sensitivity | 79 | 82 |
| Specificity | | |
| *vs* CP, CH, LC | 93 | 85 |
| *vs* CP | 86 | 76 |
| *vs* CH, LC | 95 | 88 |

CP, chronic pancreatitis; CH, chronic hepatitis; LC, liver cirrhosis. Cut-off levels: CA242, $30\,U\,ml^{-1}$; CA19-9, $37\,U\,ml^{-1}$.

**Table II** Positive ratio for CA242 and CA19-9 in pancreatic cancer by TMN staging (UICC)

| | *Cut-off ($U\,ml^{-1}$)* | *Stage I* (n = 17) (%) | *Stage II* (n = 9) (%) | *Stage III* (n = 20) (%) | *Stage IV* (n = 43) (%) |
|---|---|---|---|---|---|
| *Cut-off level from normal subjects* | | | | | |
| CA242 | 30 | 41 | 89 | 85 | 93 |
| CA19-9 | 37 | 47 | 78 | 85 | 91 |
| *Cut-off level at 90% specificity for CP, CH, LC* | | | | | |
| CA242 | 26 | 41 | 89 | 90 | 93 |
| CA19-9 | 47 | 35 | 78 | 85 | 91 |
| *Cut-off level at 90% specificity for CP* | | | | | |
| CA242 | 35 | 41 | 89 | 80 | 88 |
| CA19-9 | 80 | 29 | 78 | 70 | 88 |
| *Cut-off level at 90% specificity for CH, LC* | | | | | |
| CA242 | 23 | 47 | 89 | 90 | 93 |
| CA19-9 | 42 | 35 | 78 | 85 | 91 |

CP, chronic pancreatitis; CH, chronic hepatitis; LC, liver cirrhosis.

**Figure 5** Correlations between serum levels of the two markers in pancreatic cancer (a) and benign diseases (b).

**Figure 6** Influence of Lewis blood group system on serum CA242 and CA19-9 levels in 65 normal subjects. Determination of Lewis blood group was performed by haemagglutination test. *$P<0.05$ (ANOVA).

*Influence of Lewis blood group system on serum levels in normal subjects*

As shown in Figure 6, the serum CA242 levels in normal subjects were influenced by the Lewis blood group system, as was the serum CA19-9 level. Among the three phenotypes, a significantly lower level was found in Le(a−b−) and Le(a−b+) groups than in the Le(a+b−) group.

### Discussion

In this study, we confirmed that CA242 is a more useful marker than CA19-9 for detecting curable cases of pancreatic cancer among outpatients on their first visit, because of the similar sensitivities for overall cases and cases of early-stage (stage I) pancreatic cancer, and the higher specificity resulting from the less frequent and lower elevation in benign diseases. These results were similar to previous reports (Kuusela et al., 1991; Nilsson et al., 1992; Pasanen et al., 1992) and superior to other studies (Banfi et al., 1993; Rothlin et al., 1993). Although CA19-9 has been widely used as a tumour marker for the diagnosis of pancreatic cancer, some clinical drawbacks have been raised concerning false-negative results in many patients with localised pancreatic cancer (Steinberg et al., 1986; Malesci et al., 1987; Frebourg et al., 1988; Kawa et al., 1990), false-positive results in patients with benign diseases, especially chronic pancreatitis (Schmigel et al., 1985; Tatsuta et al., 1985; Malesci et al., 1987; Kawa et al., 1990) and liver diseases (Jalanko et al., 1984; Steinberg et al., 1986; Kawa et al., 1990; Kobayashi et al., 1991) and false-negative results in cancer patients with Lewis-negative phenotype (Hirano et al., 1987; Margaret et al., 1987; Kawa et al., 1991).

The clinical usefulness of CA19-9 in this study was similar to that reported previously, supporting the validity of the present study. The sensitivity of CA242 for pancreatic cancer was similar to that of CA19-9, and CA242 has been reported to be expressed on pancreatic cancer tissues to a similar extent as CA19-9 (Haglund et al., 1989). However, no relationship was observed between the serum levels of the two markers in this study. As suggested previously (Johanson et al., 1991a; Kuusela et al., 1991), the two structures may be different. The overall sensitivity of CA242 in other malignant diseases is lower than that of CA19-9. However, for other malignancies, more useful diagnostic tools than tumour markers can be used. Therefore, these disadvantages are negligible in the clinical use of this marker. On the other hand, CA242 gave better results in colorectal cancer than CA19-9, which is in agreement with previous findings (Kuusela et al., 1991; Nilsson et al., 1992). Its usefulness was more apparent at cut-off levels corresponding to 90% specificity. Moreover, CA242 has been reported to be useful in the diagnosis of early-stage colorectal cancer (Dukes' A and B) and a valuable complement to CEA (Kuusela et al., 1991; Nilsson et al., 1992). In hepatoma, a significant difference in the positive rate was noted between the two markers. CA19-9 is expressed not on hepatoma cells but on bile duct cells. The mechanism of serum elevation of CA19-9 is considered to be cholestasis or damage to the bile duct cells (Kobayashi et al., 1991). Although the exact mechanism of this elevation has not been elucidated, CA242 may be only slightly affected by these abnormal conditions, which was further demonstrated by the results obtained in the patients with benign obstructive jaundice.

We have confirmed that CA242 is as useful as CA19-9 in the diagnosis of stage I pancreatic cancer, including curative resectable disease, using the cut-off level obtained from normal subjects. By using cut-off levels corresponding to 90% specificity for various benign conditions, CA19-9 was falsely negative in some patients with stage I tumour, although the sensitivities of the two markers overall were similar. Resectability is considered to be an important predictor of the prognosis (Saito, 1990), and CA19-9 has been reported to be useful in the diagnosis of 50–79% of patients with resectable pancreatic cancer (Sakahara et al., 1986, Kawa et al., 1990; Kobayashi et al., 1991). As with CA19-9, CA242 has also been reported to be positive in half of patients with resectable disease (Kuusela *et al*., 1991). However, it may be clinically impractical to search for early pancreatic cancer such as stage I tumours more extensively using a tumour marker alone, and it is necessary to develop a screening system combined with other methods, such as ultrasonography, to improve the usefulness of the early diagnosis.

The major advantage of CA242 over CA19-9 is its higher specificity resulting from the less frequent and only slight elevation in the serum level in patients with chronic pancreatitis, benign liver diseases and benign obstructive jaundice, which was confirmed by the present study and previous reports (Johansson *et al*., 1991*b*; Kuusela *et al*., 1991; Nilsson *et al*., 1992; Pasanen *et al*., 1992). The differential diagnosis of pancreatic cancer from chronic pancreatitis is sometimes difficult. In patients with liver diseases, false-positive elevation of CA19-9 is frequently on the first visit to the clinic (Frebourg *et al*., 1988). Accordingly, the good discrimination provided by CA242 will avoid unnecessary further examination of the patient. CA19-9 has been demonstrated to be expressed on the duct cells of the pancreas and bile duct cells of the liver (Arends *et al*., 1983; Kobayashi *et al*., 1991) and secreted in the pancreatic juice and bile in both healthy subjects and patients with pathological conditions (Schmiegel *et al*., 1985). In benign diseases, the antigen is considered to be released into the circulation by stagnation of pancreatic juice, cholestasis or damage to the pancreatic and bile duct. However, tissue expression of CA242 in the normal pancreas and in chronic pancreatitis is reported to be similar to that of CA19-9 (Haglund *et al*., 1989). Therefore, it is uncertain why CA242 is less influenced by these conditions. A notable characteristic of CA242 is that its serum level is scarcely affected by cholestasis, which was demonstrated by less frequent and slight elevation in sera of patients with benign obstructive jaundice. The clinical results obtained in benign liver diseases may also be associated with this characteristic. Further studies, including immunohistochemistry, are necessary to clarify the exact mechanism.

In this study, the CA242 assay system was shown to be influenced by the Lewis blood group system, as in the CA19-9 assay. This drawback is considered to be attributable to the C241 catcher antibody of the assay system, because the C241 monoclonal antibody has almost the same epitope specificity as the NS19-9 monoclonal antibodies used in the CA50 and CA19-9 assay systems, whereas the C50 antibody also recognises sialylated lact-*N*-tetraose to a small degree (Nilsson *et al*., 1985; Johansson *et al*., 1991*a*). In a previous study, we confirmed that the plasma expression of CA50 is similar to that of CA19-9 with respect to Lewis blood cell status (Kawa *et al*., 1991). We also found that Lewis-negative patients constituted one-third of the CA19-9-negative patients, and the Dupan-2 assay provides a complementary method for these patients (Kawa *et al*., 1991). Accordingly, for CA242-negative patients who are suspected to have pancreatic cancer, an additional Dupan-2 assay is recommended.

In conclusion, CA242 is superior to CA19-9 in the diagnosis of pancreatic cancer because of its higher specificity, and it may be useful in the screening of localised or resectable tumours.

This work was supported by a Grant-in-Aid from the Ministry of Health and Welfare of Japan. Thanks are due to Syusuke Kitawada for his technical assistance.

### References

ARENDS, J.W., VERSTYNE, C., BOSMAN, F.T., HILGERS, J. & STEPLEWSKI, Z. (1983). Distribution of monoclonal antibody-defined monosialoganglioside in normal and cancerous human tissues: immunoperoxidase study. *Hybridoma*, **2**, 219-229.

BANFI, G., ZEBRI, A., PASTORI, S., PAROLINI, D., DI CARLO, V. & BONINI, P. (1993). Behavior of tumor markers CA19-9, CA195, CAM43, CA242, and TPS in the diagnosis and follow-up of pancreatic Cancer. *Clin. Chem*., **39**, 420-423.

DEL VILLANO, B.C., BRENNAN, S., BROCK, P., BUCHER, C., LIU, V., McCLURE, M., RAKE, B., SPACE, S., WESTRICK, B., SCHOEMAKER, H. & ZURAWSKI, Jr, V.R. (1983). Radioimmunometric assay for a monoclonal antibody-defined tumor marker, CA19-9. *Clin. Chem*., **29**, 549-552.

FREBOURG, T., BERCOFF, E., MANCHON, N., SENANT, J., BASUYAU, J.P., BRETON, P., JANVRESSE, A., BRUNELLE, P. & BOURREILLE, J. (1988). The evaluation of CA19-9 antigen level in the early detection of pancreatic cancer. *Cancer*, **62**, 2287-2290.

HAGLUND, C., LINDGREN, J., ROBERTS, P.J., KUUSELA, P. & NORDLING, S. (1989). Tissue expression of tumour-associated antigen CA242 in benign and malignant pancreatic lesions. A comparison with CA50 and CA19-9. *Br. J. Cancer*, **60**, 845-851.

HOMMA, T. & TSUCHIYA, R. (1991). The study of the mass screening of persons without symptoms and of the screening of outpatients with gastrointestinal complaints or icterus for pancreatic cancer in Japan, using CA19-9 and elastase-1 or ultrasonography. *Int. J. Pancreatol*., **9**, 119-124.

HIRANO, K., KAWA, S., OGUCHI, H., KOBAYASHI, T., YONEKURA, H., OGATA, H. & HOMMA, T. (1987). Loss of Lewis antigen expression on erythrocytes in some cancer patients with high serum CA19-9 levels. *J. Natl Cancer Inst*., **79**, 1261-1268.

JALANKO, H., KUUSELA, P., ROBERTS, P., SIPPONEN, P., HAGULUND, C. & MAKELA, O. (1984). Comparison of a new tumor marker, CA19-9 with alpha fetoprotein and carcinoembryonic antigen in patients with upper gastrointestinal diseases. *J. Clin. Pathol*., **37**, 218-222.

JOHANSSON, C., NILSSON, O., BAECKSTROEM, D., JANSSON, E.L. & LINDHOLM, L. (1991*a*). Novel epitopes on the CA50-carrying antigen: Chemical and immunochemical studies. *Tumor Biol*., **12**, 159-170.

JOHANSSON, C., NILSSON, O. & LINDHOLM, L. (1991*b*). Comparison of serological expression of different epitopes on the CA50-carrying antigen, CanAg. *Int. J. Cancer*, **48**, 757-763.

KAWA, S., OGUCHI, H., TOKOO, M., KOBAYASHI, T., IMAI, H., KIYOSAWA, K., FURUTA, S., KANAI, M. & HOMMA, T. (1990). A comparative study of the clinical usefulness of CA19-9, CA50, sialyl SSEA-1 and Dupan-2 for the diagnosis of pancreatic cancer used alone or in combination. *Jpn. J. Clin. Chem*., **19**, 389-398.

KAWA, S., OGUCHI, H., KOBAYASHI, T., TOKOO, M., FURUTA, S., KANAI, M. & HOMMA, T. (1991). Elevated serum levels of Dupan-2 in pancreatic cancer patients negative for Lewis blood group phenotype. *Br. J. Cancer*, **64**, 899-902.

KOBAYASHI, T., KAWA, S., TOKOO, M., OGUCHI, H., KIYOSAWA, K., FURUTA, S., KANAI, M. & HOMMA, T. (1991). Comparative study of CA50 (time-resolved fluoroimmunoassay), Span-1, and CA19-9 in the diagnosis of pancreatic cancer. *Scand. J. Gastroenterol*., **26**, 787-797.

KOPROWSKI, H., STEPLEWSKI, Z., MITCHELL, K., HERLYN, M. & FUHNER, P. (1979). Colorectal carcinoma antigens detected by hybridoma antibodies. *Somatic Cell Genet*., **5**, 957-972.

KUUSELA, P., HAGLUND, C. & ROBERTS, P.J. (1991). Comparison of a new tumour marker CA242 with CA19-9, CA50 and carcinoembryonic antigen (CEA) in digestive tract disease. *Br. J. Cancer*, **63**, 636-640.

LINDHOLM, L., JOHANSSON, C., JANSSON, E.-L., HALLBERG, C. & NILSSON, O. (1985). An immunometric assay (IRMA) for the CA50 antigen. In *Tumor Marker Antigens*, Holmgren, J. (ed.) p. 122. Studentlitteratur: Lund, Sweden.

MALESCI, A., TOMMASINI, M.A., BONATO, C., BOCCHIA, P., BERSANI, M., ZERBI, A., BERETTA, E. & DI CARLO, V. (1987). Determination of CA19-9 antigen in serum and pancreatic juice for differential diagnosis of pancreatic adenocarcinoma from chronic pancreatitis. *Gastroenterology*, **92**, 60-67.

MARGARET, A.T., UCHIDA, E., TAKASAKI, H., DAVID, A.B., STEPLEWSKI, Z. & POUR, P.M. (1987). Relationship of carbohydrate antigens CA19-9 and Lewis antigen in pancreatic cancer. *Cancer Res*., **47**, 5501-5503.

NILSSON, O., MANSSON, J.E., LINDHOLM, L., HOLMGREN, J. & SVENNERHOLM, L. (1985). Sialyllacttetraosylceramide, a novel ganglioside antigen detected in human carcinoma by monoclonal antibody. *FEBS Lett.*, **182**, 398–402.

NILSSON, O., JOHANSSON, C., GLIMELIUS, B., PERSSON, B., PEDERSON, N.A., SANDBERG, A. & LINDHOLM, L. (1992). Sensitivity and specificity of CA242 in gastro-intestinal cancer. A comparison with CEA, CA50 and CA19-9. *Br. J. Cancer*, **65**, 215–221.

PASANEN, P.A., ESKELINEN, M., PARTANEN, K., PIKKARAINEN, P., PENTTILA, I. & ALHAVA, E. (1992). Clinical evaluation of a new serum tumor marker CA242 in pancreatic carcinoma. *Br. J. Cancer*, **65**, 731–734.

ROTHLIN, M.A., JOLLER, H. & LARGIADER, F. (1993). CA242 is a new tumor marker for pancreatic cancer. *Cancer*, **71**, 701–707.

SAITO, Y. (chairman) (1990). Pancreatic cancer registration committee of Japan pancreas society. Annual report of national registration of pancreatic cancer patients in 1990, p. 85. Japan Pancreas Society: Kobe.

SAKAHARA, H., ENDO, K., NAKAGIMA, K., NAKASHIMA, T., KOIZUMI, M., OHTA, H., HIDAKA, A., KOHNO, S., NAKANE, Y., NAITO, A., SUZUKI, T. & TORIZUKA, K. (1986). Serum CA19-9 concentration and computed tomography findings in patients with pancreatic carcinoma. *Cancer*, **57**, 1324–1326.

SCHMIGEL, W.H., KREIKER, C., EBERL, W., ARNDT, R., CLASSEN, M., GRETEN, H., JESSEN, K., KALTHOFF, H., SOEHENDRA, N. & THIELE, H.G. (1985). Monoclonal antibody defines CA19-9 in pancreatic juices and sera. *Gut*, **26**, 456–460.

STEINBERG, W.M., GELFAND, R., ANDERSON, K.K., GLENN, J., SCOTT, H.K., WILLIAM, F.S. & PHILIP, P.T. (1986). Comparison of the sensitivity and specificity of the CA19-9 and carcinoembryonic antigen assays in detecting cancer of the pancreas. *Gastroenterology*, **90**, 343–349.

TATSUTA, M., YAMAURA, H., ISHII, H., ICHII, M., NOGUCHI, S., YAMAMOTO, R. & OKUDA, S. (1985). Values of CA19-9 in the serum, pure pancreatic juice, and aspirated pancreatic material in the diagnosis of malignant pancreatic tumor. *Cancer*, **56**, 2669–2673.